# Technology Literacy in Undergraduate Medical Education: Review and Survey of the US Medical School Innovation and Technology Programs

**DOI:** 10.2196/32183

**Published:** 2022-03-31

**Authors:** Judy Jiaqi Wang, Rishabh K Singh, Heather Hough Miselis, Stephanie Nicole Stapleton

**Affiliations:** 1 Department of Medicine Boston University School of Medicine Boston, MA United States; 2 Department of Family Medicine Boston University School of Medicine Boston Medical Center Boston, MA United States; 3 Department of Emergency Medicine Boston University School of Medicine Boston Medical Center Boston, MA United States

**Keywords:** curricular development, medical innovation, medical technology, student engagement

## Abstract

**Background:**

Modern innovations, like machine learning, genomics, and digital health, are being integrated into medical practice at a rapid pace. Physicians in training receive little exposure to the implications, drawbacks, and methodologies of upcoming technologies prior to their deployment. As a result, there is an increasing need for the incorporation of innovation and technology (I&T) training, starting in medical school.

**Objective:**

We aimed to identify and describe curricular and extracurricular opportunities for innovation in medical technology in US undergraduate medical education to highlight challenges and develop insights for future directions of program development.

**Methods:**

A review of publicly available I&T program information on the official websites of US allopathic medical schools was conducted in June 2020. Programs were categorized by structure and implementation. The geographic distribution of these categories across US regions was analyzed. A survey was administered to school-affiliated student organizations with a focus on I&T and publicly available contact information. The data collected included the founding year, thematic focus, target audience, activities offered, and participant turnout rate.

**Results:**

A total of 103 I&T opportunities at 69 distinct Liaison Committee on Medical Education–accredited medical schools were identified and characterized into the following six categories: (1) integrative 4-year curricula, (2) facilitated doctor of medicine/master of science dual degree programs in a related field, (3) interdisciplinary collaborations, (4) areas of concentration, (5) preclinical electives, and (6) student-run clubs. The presence of interdisciplinary collaboration is significantly associated with the presence of student-led initiatives (*P*=.001). “Starting and running a business in healthcare” and “medical devices” were the most popular thematic focuses of student-led I&T groups, representing 87% (13/15) and 80% (12/15) of respondents, respectively. “Career pathways exploration for students” was the only type of activity that was significantly associated with a high event turnout rate of >26 students per event (*P*=.03).

**Conclusions:**

Existing school-led and student-driven opportunities in medical I&T indicate growing national interest and reflect challenges in implementation. The greater visibility of opportunities, collaboration among schools, and development of a centralized network can be considered to better prepare students for the changing landscape of medical practice.

## Introduction

The intersection of technology and medicine has continuously transformed health care delivery [1-3]. The medical applications of advancing technologies include the use of deep learning algorithms to power diagnostics [4], automated robotics to perform minimally invasive procedures [5], and computational genomics to inform personalized treatment plans [6]. In 2020, social distancing limitations due to COVID-19 catalyzed unprecedented developments in digital health [7-9]. From video consultation platforms to home testing kits and wearable sensors, patients have been increasingly exposed to a digitally driven health care model [10,11]. The breadth of personal health data that are available to patients is larger than ever before [12,13]. However, physicians are facing an increasing need to guide patients in correctly interpreting these data as well as communicate relevant implications of technology to patients. Moreover, technology literacy in medicine, that is, a basic understanding of how new technologies work and how they can be integrated into more patient-centered and efficient health care delivery systems, may allow for more effective interdisciplinary collaboration with experts in other fields to address clinical needs in innovative ways [14,15].

No matter the objective of an individual physician, speaking the language of technology should be learned during undergraduate medical education—the earliest years of one’s training prior to the completion of an MD degree [16-18]. Some US medical schools have begun to approach the integration of technology into medical education [19-23]. However, a prior study of formal curricular programs in innovation and entrepreneurship demonstrated the lack of any formal competency models or frameworks among institutions working on this challenge [24]. Historically, medical schools have been able to adapt to health care workforce needs by providing students in training with new areas of knowledge. For example, recognizing that a patient’s health is part of a broader social and environmental context facilitated the integration of behavioral and social sciences into medical education. These changes were aimed at enabling students to better understand epidemiology, mental health, and social determinants of health [25,26]. Although integrations like these are still being refined, they can offer an implementation framework that new curricular developments can follow. A remaining challenge will be developing consensus on standards for teaching students about emergent technology. Discussions about clinical applications and implementation are somewhat speculative, as there are less supporting data than what physicians are accustomed to, and requirements differ based on location and specialty.

Medical education has historically had to balance the need for standardization with the benefits of ingenuity and diverse methodologies [27]. Due to the novelty of technology integration, it may be premature to pursue standardization before understanding the approaches that have been tried and the outcomes that they have produced. Herein, we identify and analyze the innovation and technology (I&T) opportunities available at US allopathic medical schools and discuss thematic trends to support the future development of I&T curricula. Compared to the traditional definition of innovation and entrepreneurship, which largely focuses on business and economics, we concentrated on I&T. Our analysis provides a more expansive view on the diverse formats of learning opportunities, including formal curricula as well as extracurricular electives and initiatives. This study aims to quantify and detail the existing I&T opportunities available to medical students at US medical schools to provide insight for future curricular development directions.

## Methods

The data collection process consisted of a combination of public internet searches and the collection of survey responses from student organizations across the country. Surveys were conducted in June 2020 and asked for objective information, including club characteristics, types of activities, and target audiences.

### Ethics Approval

Since no individual information or opinions were collected, this study did not meet the requirements for a human subject review, per our institutional review board’s protocol.

### Review of Current Programs

An internet search of all Liaison Committee on Medical Education–accredited US allopathic medical schools [28] was conducted to identify any relevant curricular and extracurricular programs that were offered. The key search terms were *medical education*, *technology*, *engineering*, *innovation and entrepreneurship*, *curriculum*, and *student activities/organizations*. The inclusion criteria were defined as (1) programs officially sanctioned by a medical school (ie, programs that have been recognized by school administrations and other publicly affiliated sources) and (2) programs that mentioned at least 1 of the following in their mission statement: (1) applying engineering research and existing technologies in medicine or (2) inventing and designing technological solutions in medicine. The exclusion criteria included programs without a significant technical or innovative component. These programs may (1) have a primary focus on other topics, such as business, economics, leadership, health policy, and health information management; (2) include a scholarly component on any topic of choice but do not provide a specific focus on I&T; and (3) be doctorate of medicine and philosophy (MD-PhD) programs that undergo a separate application and admission process.

### Survey on Student-Led Initiatives

The initial abstraction of public data indicated a lack of organized and publicly available information on student-led I&T organizations and activities. We designed a short, 9-question survey for student groups by using the web-based program Typeform (Typeform SL). The survey was sent electronically to all identified school-affiliated I&T groups whose contact information was publicly available. The survey consisted of 8 total questions that inquired about the (1) founding year, (2) thematic focus, (3) target audience, (4) activities offered, and (5) participant turnout rate. The responses collected contained only objective information and involved no subjective data. Recorded data were securely stored in a protected spreadsheet that was exported from Typeform.

### Data Analysis

The data analysis included both aggregated data from the internet search and completed survey responses. Programs that met the inclusion and exclusion criteria were analyzed and classified into 6 categories based on program characteristics. The geographic locations of programs were noted for regional relationships. Survey results and publicly available information, either from the clubs’ own websites or from the schools’ student activity websites, were synthesized. A thematic analysis was performed and included the following information about each program: the number years since its founding, its mission, its target audience, events and activities, and the medical student turnout rate. A statistical analysis was conducted on survey data by using SPSS version 26 (IBM Corporation) for macOS. A chi-square test of independence was performed for any associations between student-led initiatives and other curricular opportunities.

## Results

### Review of Current Programs

**Figure 1:**
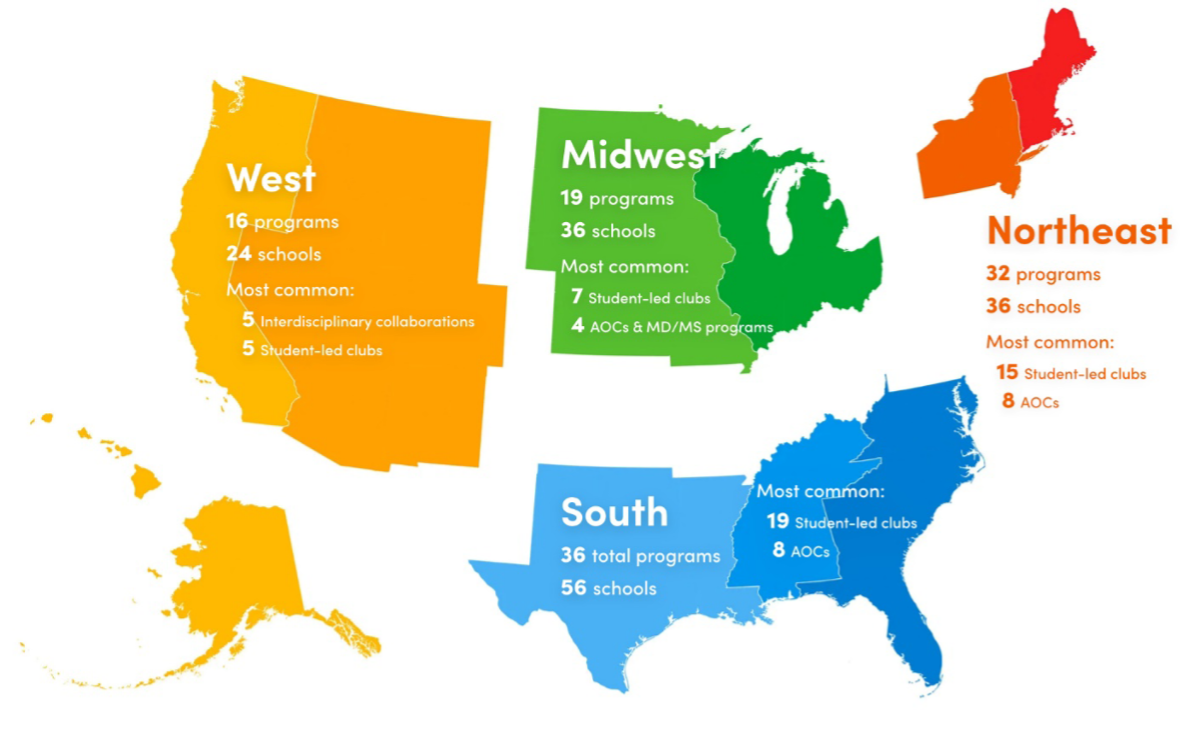
A map representation of innovation and technology programs across the major geographical regions based on the US Census. AOC: area of concentration.

Our investigation of existing programs found varying degrees of curricular integration and various durations and target audiences. A total of 103 programs at 69 distinct schools were identified to have at least 1 program that met our inclusion and exclusion criteria. Further, 6 categories were determined based on the level of administrative and student involvement of these programs (Table 1). Programs were further analyzed by geographical region (Figure 1 and Table 2). The highest ratios of the number of available programs to the number of medical schools were found in the northeast (32 programs to 36 schools; ratio: 0.89) and west (16 programs to 24 schools; ratio: 0.67). The regional subdivision with the highest program density was New England (13 programs to 10 schools; ratio: 1.30). Texas offered the greatest number of programs (8 programs to 12 schools; ratio: 0.67), followed by California (7 programs to 13 schools; ratio: 0.54) and New York (7 programs to 15 schools; ratio: 0.47). Interestingly, 16 states were identified as having 0 I&T programs available to students at their medical schools, and 14 of these states have only 1 or 2 allopathic medical schools. Further, 12 states offer more than 3 programs, with Rhode Island having the greatest number of programs per medical school (3:1 ratio).

Student-led clubs and initiatives were the most common type of opportunity available to students, representing 44.7% (46/103) of the total programs. Curricular tracks or areas of concentration were the next most common type (21/103, 20.4%), followed by interdisciplinary collaborations (14/103, 13.6%), dual degree programs in a related field (12/103, 11.7%), and noncredited elective courses (6/103, 5.8%). Of note, there are 4 special programs with a 4-year integrated curriculum (4/103, 3.9%). Table 3 shows that interdisciplinary collaborations were the only type of program that was significantly associated with the presence of student initiatives (*P*=.001; *χ*^2^_1_*=*10.6).

**Table 1:** The six identified innovation and technology program categories and descriptions of each category.

Category	Description of category	Number of programs (N=103)
4-year integrated programs	The programs exhibit longitudinal themes that are integrated across all 4 years. Admission into each program is separate from admission into the general MD degree program. Other shared characteristics include a graduating project requirement and significant accompanying research involvement. Table 3 provides a more comprehensive analysis of these programs.	4
MD/MS dual degree programs	Facilitated, and often accelerated (5 years or fewer), dual degree programs offering MS degrees in biomedical engineering or health technology.	12
Interdisciplinary collaborations	Institutes and incubators aimed at encouraging collaboration across different schools within the greater institution.	14
Tracks or areas of concentration	The programs extend over multiple semesters, with final completion being noted in the dean’s letter or official transcript. Many require 1 or more courses and a research component to supplement the regular medical curriculum.	21
Noncredited elective courses	Semester-long courses that are available to medical students for enrichment purposes. They are not credited or noted on the official transcript.	6
Student-led clubs	Student-run organizations that host regular events for the student body.	46

**Table 2:** Overview of innovation and technology programs at accredited US allopathic medical schools.

Characteristic	West region^a^	Midwest region^b^	Northeast region^c^	South region^d^	All regions
4-year integrated programs, n	1	1	1	1	4
MD/MS dual degree programs, n	3	4	1	4	12
Interdisciplinary collaborations, n	5	2	4	3	14
Concentration tracks or areas of concentration, n	1	4	8	8	21
Noncredited elective courses, n	1	1	3	1	6
Student-led clubs, n	5	7	15	19	46
Total programs, n	16	19	32	36	103
Total schools, n	24	36	36	57	153
Ratio of the number of programs to the number of schools	0.67	0.53	0.89	0.63	0.67

^a^States per region: Washington, Oregon, California, Montana, Idaho, Wyoming, Nevada, Utah, Colorado, Arizona, and New Mexico.

^b^States per region: North Dakota, South Dakota, Nebraska, Kansas, New Mexico, Iowa, Missouri, Wisconsin, Illinois, Michigan, Indiana, and Ohio.

^c^States per region: New York, Pennsylvania, New Jersey, Vermont, New Hampshire, Maine, Massachusetts, Connecticut, and Rhode Island.

^d^States per region: Oklahoma, Texas, Arizona, Louisiana, Mississippi, Alabama, Tennessee, Kentucky, West Virginia, Virginia, Maryland, Delaware, North Carolina, South Carolina, Georgia, and Florida.

**Table 3:** Associations among program categories based on the existence of student initiatives.

Program			Presence of student-led clubs							Chi-square (*df*)			*P* value
			Yes, n		No, n		Total, n						
**4-year integrated program**										—^a^			.58^b^
	Yes	2		2		4							
	No	44		105		149							
	Total	46		107		153							
**Concentration track or area of concentration**										1.4 (1)			.23
	Yes	9		13		22							
	No	37		94		131							
	Total	46		107		153							
**Noncredited elective course**										—			.37^b^
	Yes	3		3		6							
	No	43		104		147							
	Total	46		107		153							
**MD/MS dual degree program**										0.2 (1)			.69
	Yes	3		9		12							
	No	43		98		141							
	Total	46		107		153							
**Interdisciplinary collaboration** ^c^										10.6 (1)			.001
	Yes	10		5		15							
	No	36		102		138							
	Total	46		107		153							

^a^Not available.

^b^Due to the small sample size, we used the *P* value of a Fisher exact test instead of a chi-square test.

^c^Significant at the *P*<.05 level.

### Survey on Student-Led Initiatives

#### Summary of Survey Results

Of the 46 total student groups, 33 had publicly available contact information and were invited to complete the survey through email. We recorded 15 completions, indicating a 45% (15/33) response rate. The results are summarized in Multimedia Appendix 1.

#### Age Since Founding

**Figure 2:**
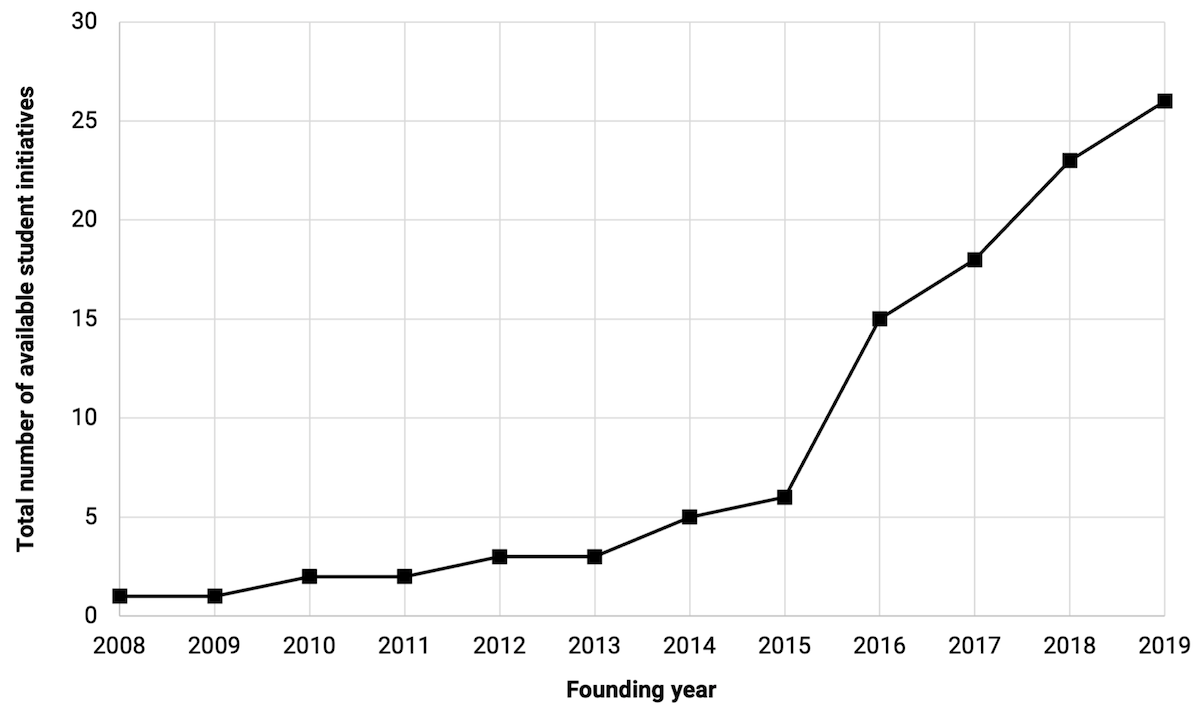
Student-led initiatives sorted based on the founding year. Founding years were either self-reported on our survey or determined based on publicly available information on medical school websites and internet archives.

The results from the survey and publicly available information yielded a total of 26 known founding years. Of the 26 student-led initiatives, 20 (77%) were founded in or after 2016, and 8 (31%) were founded in or after 2018. Figure 2 illustrates the chronological growth of these initiatives.

#### Mission

Among the 15 surveyed organizations with completed responses, student groups’ goals included “starting and running a business in healthcare” (13/15, 87%), “medical devices” (12/15, 80%), “helping students under the challenges associated with bringing ideas to market” (11/15, 73%), and “digital health” (10/15, 67%). A word cloud of club mission statements showed that *technology* (39 instances), *innovation* (38 instances), and *medicine* (30 instances) were the most common words mentioned (Multimedia Appendix 2).

#### Activities and Events

Talks hosted by either biotechnology and health industry representatives or faculty and physician speakers are the most common form of activity for student groups (13/15, 87%). Other commonly offered activities include “collaboration with schools of other disciplines” (11/15, 73%) and “connecting students to opportunities & resources” (11/15, 73%).

#### Turnout Rate and Audience

Of the 15 surveyed organizations, 12 (80%) indicated that >10 people routinely attended events. Of these 12 groups, 5 (42%) reported the attendance of between 26 and 50 people, and 1 (8%) reported the attendance of between 51 and 75 people. The events mostly targeted medical students in preclinical years (groups: 13/15, 87%) and graduate students (groups: 10/15, 67%). A minority of organizations (groups: 5/15, 33%) directly involve medical students in clerkship years, resident physicians, attending physicians, and engineering faculty. “Career pathways exploration for students” was the only type of activity that was significantly associated with a high event turnout rate of >26 students per event (*P*=.03; odds ratio 0.38, 95% CI 0.15-0.92).

## Discussion

### Current State of I&T Programs

We found a total of 103 officially sanctioned I&T programs that were available to medical students at the time of this study. These programs span 6 levels of curricular integration, ranging from student-led initiatives to fully integrated MD degree curricula. Geographically, the highest concentration of programs per school are in the northeastern and western regions, particularly in states with a high number of medical schools that highly engage with technology industries [29]. One example of a fully integrated program is EnMed—a tripartite collaboration among Texas A&M’s College of Engineering, College of Medicine, and Houston Methodist Hospital—which integrates “innovation rotations” with researchers, collaborators, and industry partners in the medical technology field within a 4-year MD degree program [30]. However, full curriculum integration is less common. The majority of the identified programs were student-run initiatives (46/103, 44.7%). From 2015 to 2019, the number of these initiatives has seen exponential growth, with greater than a striking 400% increase (6 groups to 26 groups). The majority of student groups emphasized the thematic focuses of health care entrepreneurship (13/15, 87%) and medical devices (12/15, 80%), which were most often supported by events hosted by industrial representatives and faculty speakers. In addition, 40% (6/15) of student groups reported having >26 attendees, demonstrating high student body engagement relative to the average national class size [31].

### Call for Action: Increased Interest in I&T Among Medical Students

New generations of medical students have strong interests in the technological advancements in medicine and consider these areas of growth to be essential to future clinical practice [32]. Prior survey studies have demonstrated a significant interest in medical technology and informatics among medical students and residents [33], particularly among those intending to pursue surgical specialties [34]. In another survey study, MacNevin et al [35] showed that 79.2% of second-year medical students were “technology ready,” indicating their propensity to use new technology. However, most students do not receive formal education or training in this area [36]. Our results suggest that students are taking initiative to fill unmet needs at their respective schools, highlighting the importance of developing I&T-based education programs as part of our call for educational reform [37].

Existing literature demonstrates both the benefits and challenges associated with student-led initiatives. There is evidence of student-run electives and journal clubs resulting in positive short-term outcomes [38-40]; however, medical schools need to focus more on equipping students with proper skills and resources for effecting long-lasting advancements [41]. One major challenge faced by student-led groups is recruiting and transitioning leadership between successive class years, which results in continuity gaps in provided activities from year to year. This lack of continuity may be addressed by medical school administrations taking more responsibility for their student-led groups and by introducing a structure that supports interdisciplinary collaboration. In fact, our analysis shows a significant correlation between interdisciplinary collaborations within students’ home institutions and turnout rates for student-led activities (*P*=.001). Students may find it easier to pursue projects and consider the future integration of innovation into their medical careers when they are able to collaborate with colleagues who have complementary skill sets, such as engineering and business skill sets [42-44]. This further reinforces the importance of administrative initiative in supporting students’ interests and activities.

### Future Directions: Challenges and Propositions

#### Geographical Barriers to External Support

Our review identifies several challenges in the implementation of I&T-focused initiatives in US allopathic medical schools. Our geographical analysis correlates the density of available programs with their proximity to biotechnology hubs, suggesting that regional economic factors and the availability of external support may be associated with students’ and faculties’ exposure to I&T outcomes, further encouraging interest and investment [45]. However, areas with a low biotechnology entrepreneurship presence may produce fewer physicians who are equipped to take advantage of new clinical developments, leading to disparities in future care delivery and suggesting the importance of developing I&T initiatives in these areas. When considering efforts for introducing technological concepts into medical education, McCoy et al [46] suggest distinguishing between information that physicians must know for daily practice and information that they should know for innovation advancement; the curricular components of such efforts should target the former, and robust extracurricular programs should target the latter. Given the geographic distribution of programs across the country, well-equipped and well-resourced institutions may act as examples for supporting and modeling curriculum development and developing best practices.

#### Needs Assessment for Curricular Development

This review identifies great variation in the types of opportunities being offered to students. Hence, gaining a better understanding of the efficacy and drawbacks of each approach is important to achieving improved outcomes, as previously proposed by Chan and Zary [47] in their review of implementing artificial intelligence in medical education. Echelard et al [48] have also proposed the implementation of new courses and rotations, mentorships, and expert invitations to medical schools. Rigorous assessments of program outcomes, such as students’ familiarity with medical technology concepts or the potential rise in student- and physician-driven inventions and start-ups from proactive institutions, may be valuable downstream end points. Analyses of what practicing physician innovators identify as their needs may result in the creation of a more balanced basis for, as well as increased student interest in, defining competencies in formal curricula. In the interim, offering track programs or ancillary degrees and certificates may help with the transition to the eventual curricular reform [49]. Bringing new technologies into everyday classrooms and clinical settings can help students familiarize themselves with novel operating skills and can foster the appreciation for innovative design and problem solving [50,51].

Future studies may benefit from using Association of American Medical Colleges data from the Curriculum Reports and FACTS data sets. The former may provide insight into which schools are currently pursuing curriculum changes, which competency criteria are receiving greater prioritization in these changes, and what types of instructional methods are being applied to implement these changes. FACTS data may provide insight into the backgrounds of medical school applicants and matriculants, which may help to determine whether increasing proportions of students with engineering or business backgrounds are associated with the rapid increase in the student-led initiatives reported in our study.

### Limitations

This study exhibits several limitations. First, it relied on publicly available information. Due to possible delays between the creation of initiatives and formal publicity on the web, as well as the inherent private nature of certain types of initiatives, our study may have missed more recent efforts. This may have resulted in an underestimation of recently founded programs, especially those from schools with less frequent website updates. However, one benefit of our approach is that we were able to provide a more accurate representation of how prospective trainees and collaborators are able to discover programs, as they are generally limited to publicly available information. Future studies can deliver surveys to individual medical schools to obtain a more accurate count of the number of I&T programs that each school offers. Additionally, the development of a centralized database of opportunities and joint conferences may facilitate greater discoverability within the medical education community.

A second limitation was the challenge of surveying student organizations through publicly available contact information. In some cases, publicly available contact information was unavailable or outdated, resulting in only 33 of the 46 identified programs being sent surveys and contributing to our survey response rate. As in all survey studies, limitations in the generalizability and inflexibility of multiple-choice responses apply to our study. Our survey may be biased toward more active student organizations who provide contact information publicly and routinely respond to inquiries. Inactive student organizations may have low levels of student engagement and few organized activities; therefore, these organizations may be underrepresented in our results. Future studies may mitigate this problem by engaging medical school activity coordinators, who may provide more recent contact information and status information on club inactivity.

### Conclusions

New technologies and innovations are transforming medicine and clinical care. Efforts in exposing students to technology and innovation in medical school will prepare students for the changing landscape of medical practice. Our review of existing opportunities indicates both the growing interest in introducing trainees to medical I&T and the current challenges in integrating formalized curricular changes. Immediate and tangible future directions include increasing the visibility of current and future opportunities, achieving greater collaboration among schools, and establishing a national competency curriculum as well as a centralized platform that interested students and educators can use to share experiences.

## Appendix

Multimedia Appendix 1 Student organization survey items and responses.

Multimedia Appendix 2 Word cloud generated from student organizations' mission statements.

## Abbreviations

I&T: innovation and technology
