# Supplementary material for: Technology Literacy in Undergraduate Medical Education: Review and Survey of the US Medical School Innovation and Technology Programs
Source: JMIR Med Educ. 2022 Mar 31;8(1):e32183. doi: 10.2196/32183 (PMC9015763; doi:10.2196/32183)
Supplement: Multimedia Appendix 1 [file mededu_v8i1e32183_app1.docx]

**APPENDIX 1: Student Organizations Survey Items and Responses**

MedTech & Innovation Student Group Survey

1. Medical School: [free text]
2. Name of Student Group: [free text]
3. Email address: [free text]
4. What year was your group founded? [free text]
5. What types of activities do you offer? (select all that apply)

*Answer choices:*

- 1. Career pathways exploration for students
  2. Talks by faculty or physician speakers
  3. Talks by biotech & health tech industry representatives
  4. Collaboration with other schools (engineering, business, public health, etc.)
  5. Hands-on workshops led by students
  6. Hands-on workshops led by faculty & professionals
  7. Guided team projects, possibly overseen by faculty mentors
  8. Hackathons, design sprints, or similar activities
  9. Group discussions about upcoming tech & clinical implications
  10. Connecting interested students to opportunities and resources
  11. Other

1. What is the typical turnout rate at the events you host?

*Answer choices:*

- 1. < 10 students
  2. 10 – 25 students
  3. 26 – 50 students
  4. 51 – 75 students
  5. > 75 students

1. Who typically comes out to these events? (select all that apply)

*Answer choices:*

- 1. Medical students in preclinical years
  2. Medical students in clerkship years
  3. Graduate students (i.e. MS, PhD)
  4. Other healthcare professional students (nursing, PA, dental, etc.)
  5. Resident physicians
  6. Attending physicians
  7. Other

1. Which of the following topics does your group focus on? (select all that apply)

*Answer choices:*

- 1. Medical devices (i.e. minimally invasive tools, diagnostic machines)
  2. Digital health (i.e. wearable sensors, apps, telemedicine)
  3. Precision medicine (i.e. personalized treatment, genomics, big data analytics)
  4. Biomaterials, tissue engineering & pharmaceuticals
  5. Starting & running a business in healthcare
  6. HelpHHelfdfdHelping students understand the challenges associated with bringing their new ideas to the market/clinic
  7. Optimizing healthcare systems & care delivery
  8. Other

Survey Response - Characteristics of Student Organizations with a Focus on Innovation and Technology

|  | n = 15 | % |
| --- | --- | --- |
| **Mission** |  |  |
| Starting & running a business in healthcare | 13 | 87 |
| Medical devices | 12 | 80 |
| Understanding challenges with bringing ideas to market | 11 | 73 |
| Digital health | 10 | 67 |
| Biomaterials & tissue engineering | 6 | 40 |
| Healthcare systems & delivery | 5 | 33 |
| Precision medicine | 3 | 20 |
| Other | 2 | 13 |
| **Types of activities** |  |  |
| Talks from biotech & health tech industry | 13 | 87 |
| Talks from faculty or guest physicians | 13 | 87 |
| Collaboration with other schools | 11 | 73 |
| Connecting interested students to opportunities & resources | 11 | 73 |
| Group discussions | 9 | 60 |
| Career pathways exploration for students | 8 | 53 |
| Guided team projects | 8 | 53 |
| Hackathons, design sprints | 8 | 53 |
| Hands-on workshops led by students | 7 | 47 |
| Hands-on workshops led by faculty & professionals | 6 | 40 |
| Other | 1 | 7 |
| **Average turnout rate** |  |  |
| < 10 students | 3 | 20 |
| 10 – 25 students | 7 | 47 |
| 26 – 50 students | 4 | 27 |
| 51 – 75 students | 1 | 7 |
| 75+ students | 0 | 0 |
| **Target audience** |  |  |
| Medical students in preclinical years | 13 | 87 |
| Medical students in clinical years | 5 | 33 |
| MS/PhD graduate students | 10 | 67 |
| Other healthcare professional students | 4 | 27 |
| Resident physicians | 0 | 0 |
| Attending physicians | 2 | 13 |
| Other | 3 | 20 |
