# Supplementary figures and images for: Technology Literacy in Undergraduate Medical Education: Review and Survey of the US Medical School Innovation and Technology Programs
Source: JMIR Med Educ. 2022 Mar 31;8(1):e32183. doi: 10.2196/32183 (PMC9015763; doi:10.2196/32183)

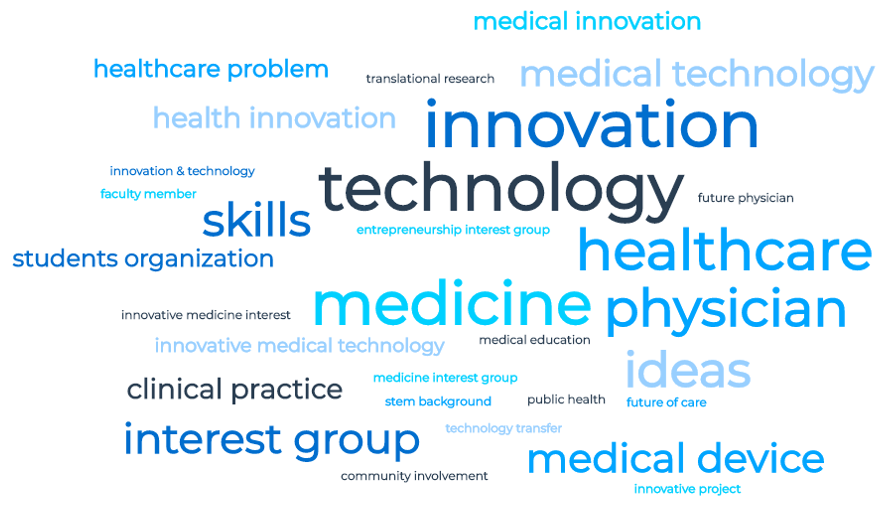

Supplement: Multimedia Appendix 2 [file mededu_v8i1e32183_app2.png]
